# Intravenous Opioid Medication with Piritramide Reduces the Risk of Pneumothorax During CT-Guided Percutaneous Core Biopsy of the Lung

**DOI:** 10.1007/s00270-024-03717-w

**Published:** 2024-04-19

**Authors:** Andrea Goetz, Florian Poschenrieder, Frederike Georgine Steer, Florian Zeman, Tobias J. Lange, Sylvia Thurn, Barbara Greiner, Christian Stroszczynski, Wibke Uller, Okka Hamer, Simone Hammer

**Affiliations:** 1 Department of Radiology, University Hospital Regensburg, Franz-Josef-Strauss-Allee 11, 93053 Regensburg, Germany; 2 Center for Clinical Trials, University Hospital Regensburg, Franz-Josef-Strauss-Allee 11, 93053 Regensburg, Germany; 3 Department of Internal Medicine II, University Hospital Regensburg, Franz-Josef-Strauss-Allee 11, 93053 Regensburg, Germany; 4 Department of Diagnostic and Interventional Radiology, Faculty of Medicine, Medical Center University of Freiburg, Hugstetter Straße 55, 79106 Freiburg, Germany

**Keywords:** Computed tomography, Biopsy, Pneumothorax, Piritramide, Analgesics, Opioid, Risk factors

## Abstract

**Purpose:**

CT-guided percutaneous core biopsy of the lung is usually performed under local anesthesia, but can also be conducted under additional systemic opioid medication. The purpose of this retrospective study was to assess the effect of intravenous piritramide application on the pneumothorax rate and to identify risk factors for post-biopsy pneumothorax.

**Materials and Methods:**

One hundred and seventy-one core biopsies of the lung were included in this retrospective single center study. The incidence of pneumothorax and chest tube placement was evaluated. Patient-, procedure- and target-related variables were analyzed by univariate and multivariable logistic regression analysis.

**Results:**

The overall incidence of pneumothorax was 39.2% (67/171). The pneumothorax rate was 31.5% (29/92) in patients who received intravenous piritramide and 48.1% (38/79) in patients who did not receive piritramide. In multivariable logistic regression analysis periinterventional piritramide application proved to be the only independent factor to reduce the risk of pneumothorax (odds ratio 0.46, 95%-confidence interval 0.24, 0.88; *p* = 0.018). Two or more pleura passages (odds ratio 3.38, 95%-confidence interval: 1.15, 9.87; *p* = 0.026) and prone position of the patient (odds ratio 2.27, 95%-confidence interval: 1.04, 4.94; *p* = 0.039) were independent risk factors for a higher pneumothorax rate.

**Conclusion:**

Procedural opioid medication with piritramide proved to be a previously undisclosed factor decreasing the risk of pneumothorax associated with CT-guided percutaneous core biopsy of the lung.

**Level of Evidence 4:**

small study cohort.

**Graphic Abstract:**

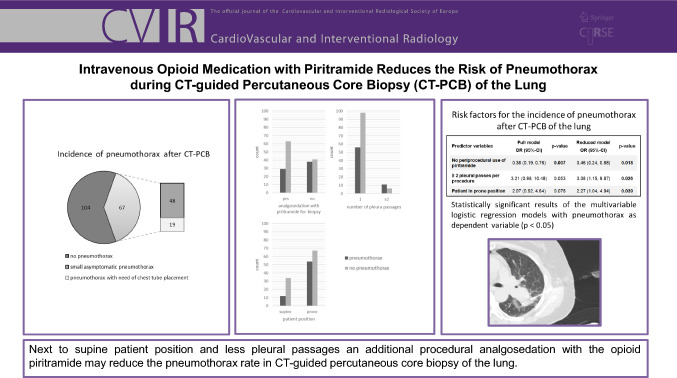

## Introduction

In the diagnostic work-up of lung lesions CT-guided percutaneous core biopsy (CT-PCB) is a well-established interventional procedure which, however, carries a considerable risk of pneumothorax. In a recent meta-analysis including 32 articles and 8,133 core biopsy procedures, the pooled pneumothorax rate was 25.3% resulting in drainage placement in 5.6% of patients [1]. Previous studies have identified different patient-, target- and procedure-related factors influencing the pneumothorax rate in CT-PCB of the lung. Still, the interventional radiologist cannot modify patient- and target-related risk factors, like presence of emphysema [2–4] or anatomic nodule location [4–8]. Various procedure-related modifications, e.g. patient position, and different post-biopsy maneuvers have been shown to be protective factors [9–12]. Only one study investigated the effect of conscious sedation on the incidence of post-biopsy pneumothorax showing no statistically significant result [13]. In contrast, based on personal, initially unsystematic individual case observation we hypothesized that procedural intravenous opioid administration could lower the risk for pneumothorax. Several effects of opioid application may be of relevance. Due to their pharmacodynamic mode of action opioid analgesics, beyond analgesia, exert an anxiolytic and sedative effect and attenuate central respiratory drive. Thereby regular, slowed and flattened breathing is facilitated. Furthermore, opioids suppress the urge to cough [14]. The sum of these effects might reduce the risk of developing pneumothorax.

The aim of this retrospective observational study was to identify patient-, target- and procedure-related risk factors for pneumothorax and to evaluate if additional opioid medication with piritramide reduces the risk of pneumothorax.

## Materials and Methods

This single center study was conducted according to the principles expressed in the Declaration of Helsinki. Institutional review board approval was obtained. The requirement for informed consent was waived for this retrospective study.

### Study Cohort

Patients were identified by means of a full-text database query of all CT-scans conducted in our tertiary care university medical center over an 11-year period using the terms „CT-guided,” “lung” and “biopsy” in the Radiological Information System (Nexus.medRIS, Version 8.42, Nexus, Villingen-Schwenningen, Germany). Inclusion criteria were technically successful CT-PCB of lung lesions. Exclusion criteria were: preexisting pneumothorax and fluid specimen aspiration (in case of lung abscess).

### Biopsy Technique and Patient Management

All patients referred to the Department of Radiology were inpatients, and senior radiologists experienced in CT-guided percutaneous interventions performed or supervised the procedures. All interventions were performed under local anesthesia. Piritramide was additionally administered intravenously at the responsible interventionalist’s discretion and determination of dosage directly after positioning the patient on the CT table and connecting the patient to a surveillance monitor measuring the heart rate and oxygen saturation rate. By administration of piritramide level 1 to level 2 of sedation and analgesia according to the American Society of Anesthesiologists definition was induced [15]. Interventions were conducted either using sequential CT guidance or CT fluoroscopy, using one of the following CT scanners: Siemens Somatom Plus 4, Siemens Somatom Sensation 16 and Siemens Somatom Definition AS (Siemens Healthcare, Erlangen, Germany). All biopsies were core biopsies performed with semi-automatic notch sample devices and coaxial technique was the preferred biopsy method at our institution.

To rule out pneumothorax 1.) CT slices focused on the level of the biopsy site at the end of the procedure and 2.) chest x-ray about 3 h after the procedure were obtained. In case of a clinically relevant pneumothorax (depending on size and clinical symptoms), a chest tube was inserted. Patients were under observation for at least one night.

### Analyzed Parameters

The analyzed data were collected by reviewing the medical records, procedural CT images and post-procedural chest x-ray images. The following patient-related data were noted: age, gender, weight and height with body mass index, history of smoking, previous thoracic surgery or tuberculosis, major comorbidities concerning the lungs and airways (asthma, chronic obstructive pulmonary disease and emphysema) and sleep apnea.

Concerning the target lesion the following parameters were recorded: location, size, pleural contact (yes/pleural tag/no), distance to the parietal pleura (measured along the needle path from the parietal pleura to the needle insertion point of the lesion), proximity to the diaphragm (defined by concomitant visibility of the diaphragm on a transversal CT slice at the level of the target lesion), cavitation (if present, wall thickness), presence of emphysema along the needle trajectory and histopathological diagnosis.

The following procedure-related data were noted: patient positioning (prone vs. supine vs. lateral), intravenous opioid administration (yes/no; dosage), caliber of the outmost biopsy needle (summarized to larger or equal to 18G and smaller or equal to 19G) and the number of pleural passages per procedure (including crossing of pleural fissures).

Complications were documented according to the standards of practice guidelines of the Cardiovascular and Interventional Radiological Society of Europe (CIRSE) [16]. In case of pneumothorax the largest distance of retraction of pulmonary surface was measured. Pneumothorax was classified into (1) mild asymptomatic and (2) symptomatic requiring chest tube placement (duration of chest tube therapy was documented). Nausea and vomiting as potential adverse effect of piritramide were documented.

### Statistical Analysis

Continuous variables are presented as mean ( ± standard deviation, SD) and categorical variables as absolute and relative frequencies. For single factor analysis of/to test for differences between continuous variables the unpaired Student t test was used, and for single factor analysis of/to test for differences between categorical data the Pearson’s chi-square test was applied. To assess risk factors for developing a pneumothorax, univariate logistic regression models were calculated in a first step. Afterwards, 8 variables with clinical relevance, which might affect the risk for pneumothorax (proximity of the target lesion to the diaphragm, emphysema along the needle trajectory, distance of the target lesion to the pleural surface) or with statistical significance in the univariate logistic regression model were selected and added to a multivariable model (full model). Since the number of events per variable is quite low ( < 10) in this model, a reduced multivariable logistic regression model was calculated, including only significant variables after using a forward selection model of the 8 pre-selected variables. Due to the smaller number of variables in the model, these have a higher power to show a significant effect. Both the full and the reduced model were calculated including 167 patients who had complete data for all considered variables. Four patients were not included in the multivariable analysis because the biopsy was performed in lateral position. For all logistic regression models, odds ratios (OR) and corresponding 95% confidence intervals (95%-CI) are reported as effect estimates. A *p* value < 0.05 was considered as statistically significant. All analyses were performed using IBM SPSS Statistics 25 (IBM, Armonk, New York, USA).

## Results

**Fig. 1:**
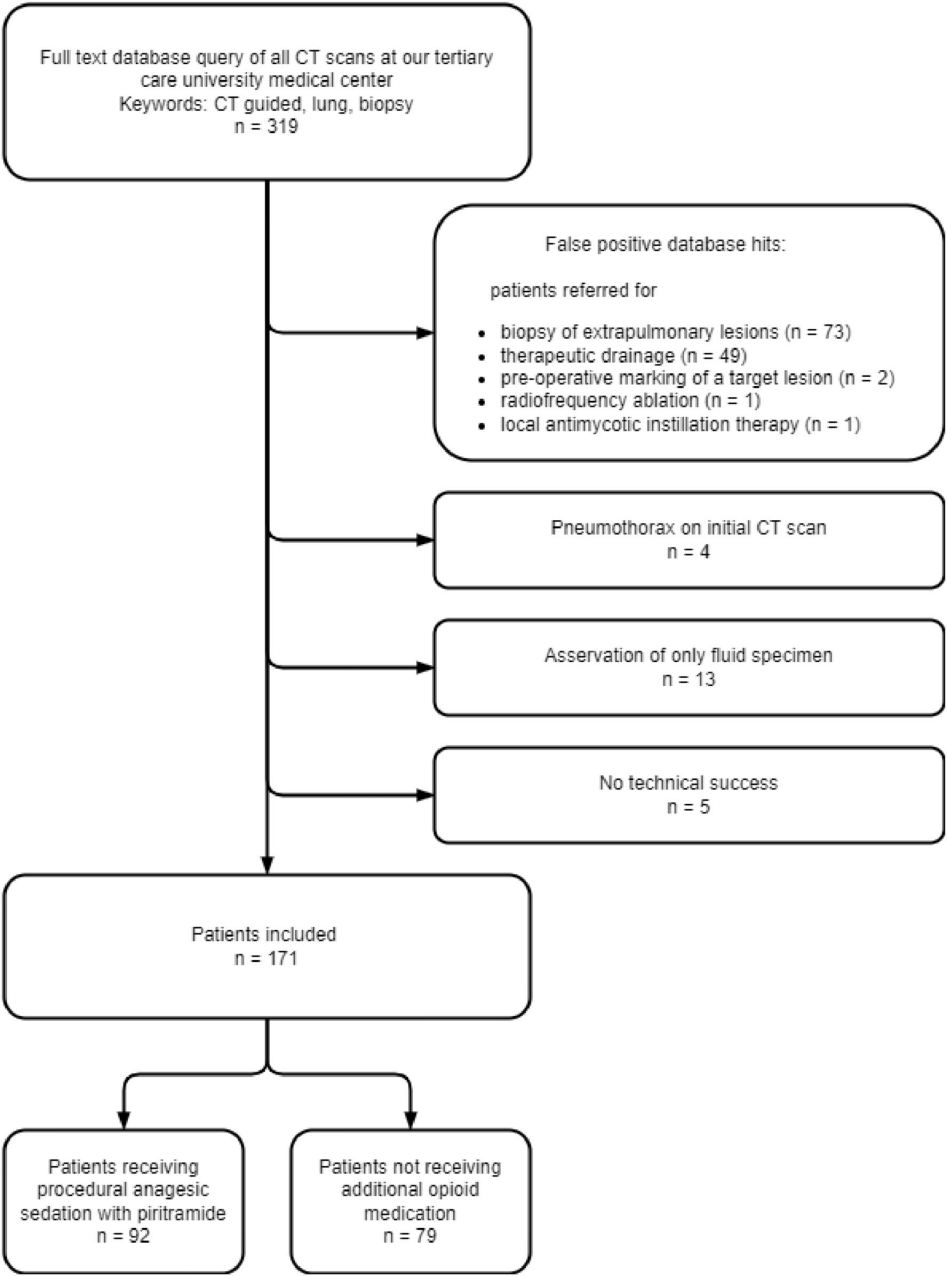
Flowchart of the study cohort

The full-text database query identified 319 patients whose records were reviewed. Figure 1 shows the flowchart of the study cohort. Finally, 171 patients were included in the study. The study cohort included 117/171 men (68%) and 54/171 women (32%); the mean patient age was 65.7 ± 12.0 years (range 23–86 years). The general patient characteristics are listed in Table 1. 111/171 interventions (64.9%) were conducted under sequential CT-guidance and 60/171 interventions (35.1%) under fluoroscopic CT-guidance. Patients were positioned in the supine position in 46/171 cases (26.9%) and in the prone position in 121/171 cases (70.8%). 4/171 patients (2.3%) underwent the procedure in lateral position, due to small group size this group was not considered separately in the statistical analysis. 98.2% (168/171) of the biopsies were performed in coaxial technique using coaxial needle calibers ranging from 17G/18G to 19G/20G. The remaining 3/171 procedures (1.8%) were direct biopsies (needle caliber was 18G or 20G) of the target lesion without using an insertion cannula. 53.8% of the patients (92/171) received periprocedural intravenous piritramide with doses ranging from 3.75 to 20 mg (mean dose 7.5 ± 3.1 mg). In one case (1/92) severe nausea (without vomiting) occurred.

**Table 1:** Patient characteristics and univariate analysis of patient-related pneumothorax risk factors

	Total *n* = 171	Pneumothorax group *n* = 67	Non-pneumothorax group *n* = 104	OR (95%-CI)	*p* value
Age (years)*	65.7 ± 12.0	67.0 ± 9.8	64.8 ± 13.3	1.02 (0.99,1.04)	0.247
Gender					
Male	117	46 (39.3)	71 (60.7)	1.02 (0.53,1.97)	0.958
Female	54	21 (38.9)	33 (61.1)	–	
Body mass index (kg/m^2^)*	26.5 ± 5.0	26.9 ± 4.8	26.2 ± 5.2	1.03 (0.95,1.11)	0.479
History of smoking**					
Yes	103/131	42/103 (40.8)	61/103 (59.2)	1.24 (0.52,2.95)	0.628
No	28/131	10/28 (35.7)	18/28 (64.3)	–	
History of thoracic surgery					
Yes	17	4 (23.5)	13 (76.5)	0.44 (0.14,1.43)	0.173
No	154	63 (40.9)	91 (59.1)	–	
History of tuberculosis					
Yes	8	3 (37.5)	5 (62.5)	0.93 (0.21,4.02)	0.921
No	163	64 (39.3)	99 (60.7)	–	
Asthma					
Yes	2	0 (0.0)	2 (100.0)	n.c	n.c
No	169	67 (39.6)	102 (60.4)		
Chronic obstructive pulmonary disease					
Yes	48	22 (45.8)	26 (54.2)	1.47 (0.75,2.88)	0.267
No	123	45 (36.6)	78 (63.4)		
Emphysema					
Yes	23	8 (34.8)	15 (65.2)	0.81 (0.32,2.02)	0.643
No	148	59 (39.9)	89 (60.1)	–	
Sleep apnea					
Yes	8	3 (37.5)	5 (62.5)	0.93 (0.21,4.02)	0.921
No	163	64 (39.3)	99 (60.7)	–	

*T*-test for continuous variables and chi-squared test of independence for categorical data

Except where otherwise indicated data are numbers with percentages in parentheses

*OR*, odds-ratio; *CI*, confidence interval; *n.c.*, not calculable due to quasi separated data

^*^ data are mean ± standard deviation

^**^ data not available for n = 40 patients

Age and gender distribution were similar in the piritramide-receiving (65.2 ± 12.0 years, male: 64.1%) and the non-piritramide group (66.1 ± 12.2 years, male: 73.4%). 67/171 patients (39.2%) developed a pneumothorax. In 48/67 cases (71.6%) a mild asymptomatic pneumothorax (mean 1.04 ± 0.54 cm, range 0.2–2.5 cm) occurred. These pneumothoraces were treated conservatively with subsequent gradual spontaneous resolution of the pneumothorax. In 19/67 patients (28.4%) the pneumothorax was symptomatic requiring a chest tube insertion. The mean dwelling time of the chest tubes was 5 ± 3.0 days.

### Univariate Analysis of Risk Factors for Pneumothorax

**Fig. 2:**
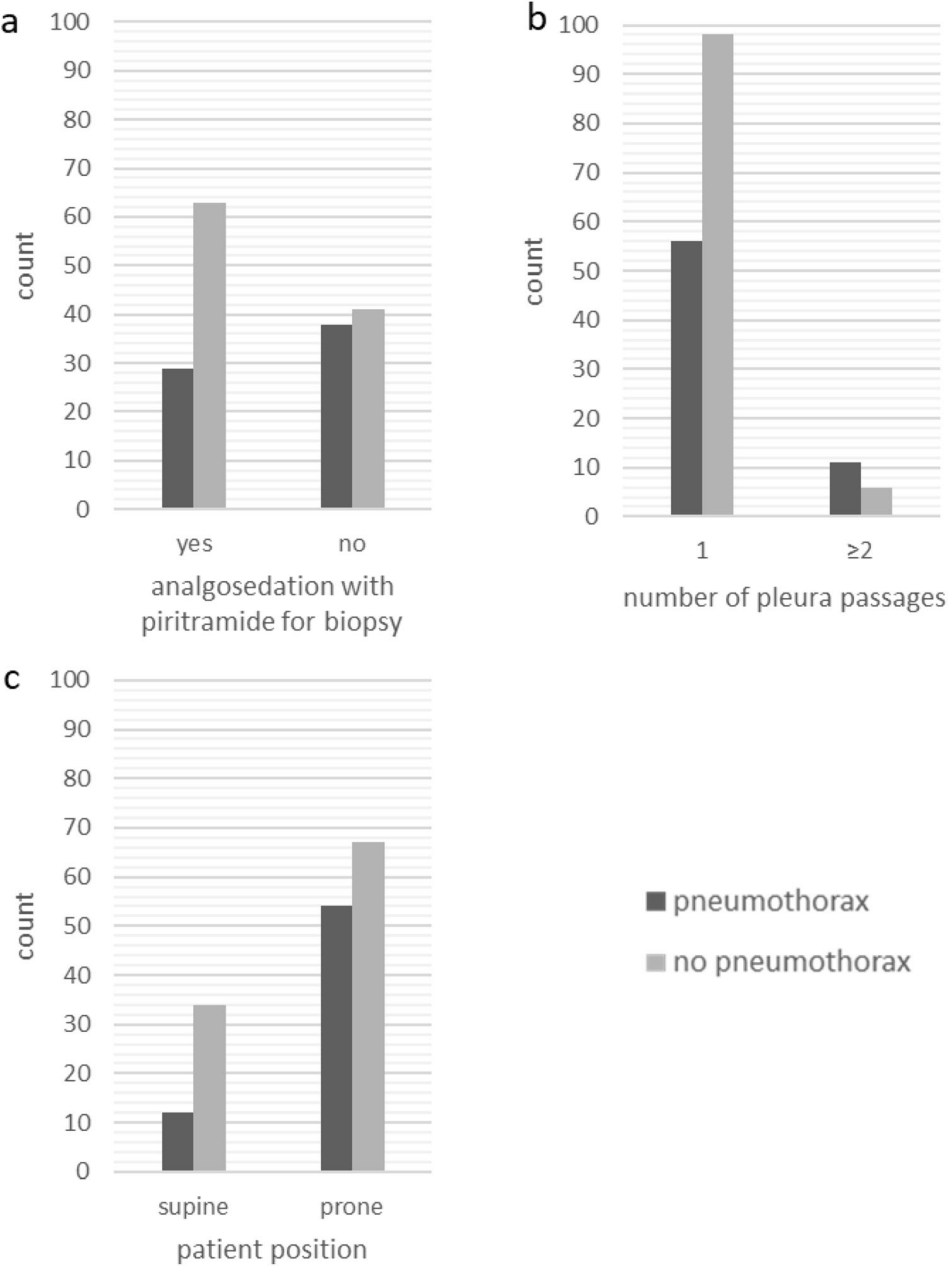
Bar graphs of incidence of pneumothorax with 3 statistically significant risk factors: **a** periprocedural analgosedation with piritramide, **b** number of pleura passages and **c** patient position

Results of univariate analysis regarding patient-related risk factors are summarized in Table 1. None of these factors exhibited a statistically significant effect on the pneumothorax rate. Table 2 lists the results of univariate analysis regarding procedure-related risk factors. A significant higher pneumothorax rate was shown in case of prone patient position (OR 2.28, 95%-CI 1.08, 4.83; *p* = 0.031), and ≥ 2 passages through the pleura during intervention (OR 3.21, 95%-CI 1.13, 9.15; *p* = 0.029), whereas the pneumothorax rate was lower in case of procedural medication with piritramide (OR 0.50, 95%-CI 0.27, 0.93; *p* = 0.028) (Fig. 2).

**Table 2:** Univariate analysis of procedure-related risk factors for pneumothorax

	Total *n* = 171	Pneumothorax group *n* = 67	Non-pneumothorax group *n* = 104	OR (95%-CI)	*p* value
Periprocedural use of piritramide					
Yes	92	29 (31.5)	63 (68.5)	0.50 (0.27, 0.93)	0.028
No	79	38 (48.1)	41 (51.9)	–	
Patient positioning*					
Prone	121	54 (44.6)	67 (55.3)	2.28 (1.08, 4.83)	0.031
Supine	46	12 (26.1)	34 (73.9)	–	
CT-guidance					
Fluoroscopic	60	23 (38.3)	37 (61.7)	0.95 (0.50, 1.80)	0.867
Sequential	111	44 (39.6)	67 (60.4)	–	
Coaxial technique					
Yes	168	67 (39.9)	101 (60.1)	n.c	0.082
No	3	0 (0.0)	3 (100.0)		
Needle caliber					
> 19G	121	46 (38.0)	75 (62.0)	0.85 (0.43, 1.66)	0.628
≤ 18G	50	21 (42.0)	29 (58.0)	–	
Number of pleura passages per procedure					
≥ 2	17	11 (64.7)	6 (35.3)	3.21 (1.13, 9.15)	0.029
1	154	56 (36.4)	98 (63.6)	–	

Chi-squared test of independence for categorical data

Data are numbers with percentages in parentheses

*OR*, odds-ratio; *CI*, confidence interval; *n.c.*, not calculable due to quasi-separated data

^*^ Data for *n* = 167 patients (in *n* = 4 cases patient position for biopsy was lateral)

None of the target-related risk factors had a statistically significant effect on the pneumothorax rate (Table 3).

**Table 3:** Univariate analysis of target-related risk factors for pneumothorax

	Total *n* = 171	Pneumothorax group *n* = 67	Non-pneumothorax group *n* = 104	OR (95%-CI)	*p* value
Size of target lesion (cm)*	171	2.56 (± 1.14)	2.99 (± 1.52)	0.79 (0.62, 1.01)	0.056
Location of target lesion					
Right upper lobe	43	15 (34.8)	28 (65.2)	0.78 (0.38, 1.61)	0.505
Left upper lobe	25	8 (32.0)	17 (68.0)	0.69 (0.28, 1.71)	0.428
Right lower lobe	49	20 (40.8)	29 (59.2)	1.10 (0.56, 2.16)	0.781
Left lower lobe	49	22 (44.9)	27 (55.1)	1.39 (0.71, 2.73)	0.333
Middle lobe	5	2 (40.0)	3 (60.0)	1.04 (0.17, 6.37)	0.970
Proximity of target lesion to diaphragm					
Yes	59	26 (44.1)	33 (55.9)	1.36 (0.72, 2.59)	0.343
No	112	41 (36.7)	71 (63.4)	–	
Pleural contact of target lesion					
Yes	125	45 (36.0)	80 (64.0)	0.69 (0.31, 1.57)	0.378
Pleural tag	17	9 (52.9)	8 (47.1)	1.39 (0.42, 4.60)	0.595
No	29	13 (44.8)	16 (55.2)	1.32 (0.59, 2.97)	0.495
Distance of target lesion to pleural surface (cm)*	171	1.10 (± 1.41)	0.80 (± 1.06)	1.22 (0.95, 1.58)	0.119
Lung emphysema along needle access course					
Yes	21	10 (47.6)	11 (52.4)	1.48 (0.59,3. 71)	0.400
No	150	57 (38.0)	93 (62.0)	–	
Cavitation of target lesion					
Yes	25	10 (40.0)	15 (60.0)	1.04 (0.44, 2.48)	0.928
No	146	57 (39.0)	89 (61.0)		
Wall thickness of cavitated target lesions (cm)**					
≤ 1.00	13	5 (38.5)	8 (61.6)	0.88 (0.18, 4.34)	0.870
> 1.00	12	5 (41.7)	7 (58.3)	–	
Histology***					
Lung cancer	81	29 (35.8)	52 (64.2)	1.05 (0.54, 2.04)	0.889
Other malignant tumor	19	7 (36.8)	12 (63.2)	1.08 (0.40, 2.93)	0.880
Inflammation	39	11 (28.2)	28 (71.8)	0.65 (0.29, 1.43)	0.285
Unspecific finding	9	5 (55.6)	4 (44.4)	2.42 (0.62–9.44)	0.202
Interstitial lung disease	4	2 (50.0)	2 (50.0)	1.87 (0.26, 13.63)	0.539
Benign tumor	1	0 (0.0)	1 (100.0)	–	–

*T*-test for continuous variables and chi-squared test of independence for categorical data

Except where otherwise indicated data are numbers with percentages in parentheses

*OR*, odds-ratio; *CI*, confidence interval

^*^ Data are mean ± standard deviation

^**^ Data for *n* = 146 patients not available

^***^ Data for *n* = 18 patients not available

### Multivariable Analysis Predicting Probability of Pneumothorax

In the full model (Table 4) the administration of piritramide was the only independent variable significantly reducing the risk for pneumothorax (OR 0.38, 95%-CI 0.19, 0.76; *p* = 0.007). In the reduced model three variables turned out to affect the risk for pneumothorax significantly (Table 4): ≥ 2 pleura passages per procedure (OR 3.38; 95%-CI 1.15, 9.87; *p* = 0.026) and prone patient position (OR 2.27; 95%-CI 1.04, 4.94; *p* = 0.039) were independent factors, which increased the risk for pneumothorax by 3.4-fold and 2.3-fold, respectively, whereas the administration of piritramide proved to be an independent factor significantly reducing the risk for pneumothorax (OR 0.46; 95%-CI 0.24, 0.88; *p* = 0.018).

**Table 4:** Multivariable logistic regression models with pneumothorax as dependent variable

Predictor variables	Full model * OR (95%-CI)	*p* value	Reduced model ** OR (95%-CI)	*p* value
Periprocedural use of piritramide	0.38 (0.19, 0.76)	0.007	0.46 (0.24, 0.88)	0.018
Proximity of target lesion to diaphragm	1.39 (0.67, 2.87)	0.371	–	–
≥ 2 pleural passages per procedure	3.21 (0.98, 10.48)	0.053	3.38 (1.15, 9.87)	0.026
Emphysema along needle access course	1.51 (0.55, 4.17)	0.428	–	–
Patient in prone position	2.07 (0.92, 4.64)	0.078	2.27 (1.04, 4.94)	0.039
No previous thoracic surgery	2.51 (0.74, 8.52)	0.140	–	–
Distance of target lesion to pleural surface	1.15 (0.85, 1.56)	0.363	–	–
Size of target lesion	0.80 (0.61, 1.04)	0.098	–	–

*OR*, odds-ratio; *CI*, confidence interval

^*^ Includes all variables with procedure-related relevance (proximity of the target lesion to the diaphragm, emphysema along the needle access course, distance of the target lesion to the pleural surface) or statistical significance in the univariate model

^**^ Forward selection model, containing only significant variables after removing non-significant variables of the full model

## Discussion

Pneumothorax is the most frequent and clinically relevant complication of CT-PCB of the lung, resulting in the necessity of chest tube placement with longer hospitalization in a substantial number of cases [1, 10, 17]. Identifying factors that might have an effect on the risk of biopsy induced pneumothorax is important in order to improve patient safety.

We conducted a retrospective analysis to assess risk and protecting factors for the induction of a pneumothorax during CT-PCB. We particularly addressed the question if intravenous piritramide medication might reduce the pneumothorax rate. Among all tested procedure-, patient- and target-related parameters multivariable analysis revealed the administration of piritramide to be the only independent variable which significantly reduced the risk for pneumothorax. Besides their analgetic and anxiolytic effect, opioids modify central respiratory drive with consecutive flattening of breathing movement and decreasing respiratory frequency. Moreover, they exhibit a central antitussive effect [14]. These pleiotropic pharmacodynamic effects increase patient’s compliance, thus reducing the extent of pleural injury, which might result in a reduced pneumothorax rate. In the literature, it has been recommended to perform CT-guided lung biopsies without sedation due to the importance of the patients’ cooperation regarding breathing instructions [18, 19]. However, in our experience intravenous piritramide medication inducing minimal to moderate sedation along with anxiolysis and analgesia does not hamper the procedure. Quite the contrary, especially the anxiolytic effect reduces patients´ discomfort and fosters patients´ cooperation. In many European countries, especially Germany, piritramide is the first-line opioid analgesic drug for the management of postoperative or posttraumatic pain [20]. Piritramide has been used for decades at our institution for periprocedural analgosedation with good experiences regarding effectiveness, safety and side effect profile. Yet the long onset time (17 min [20]) may delay the start of the biopsy. As piritramide is only approved in some European countries (e.g., Germany and Austria) but not for example in the United States of America [21], it would be interesting to investigate if the protective effect of piritramide could also be seen using similar opioid analgesics, for example fentanyl. Fentanyl (often in combination with midazolam) is also widely used for periprocedural analgosedation during CT-PCB of the lung [22] as it has a faster onset of action and a shorter duration than piritramide [20] which makes it easier to control. Covey et al. report no statistically significant difference between patients who received conscious sedation and patients who received local anesthesia only [13]. This result is not directly comparable to our results as Covey et al. investigated the usage of a different opioid (pethidine) in combination with midazolam for analgosedation.

There were two parameters which significantly increased the pneumothorax risk: ≥ 2 pleural passages and prone patient positioning. Previous studies also identified the number of pleural passages (including fissure crossing) as a risk factor [3, 4, 17]. The most likely reason is the greater damage to the pleura and alveoli. This is important for biopsy planning, fissure crossing should be avoided whenever possible.

Regarding the optimal patient position there has been some debate in the literature with opposite results. In our study cohort, the prone patient position was an independent risk factor for pneumothorax. Zhao et al. also describe the prone patient position as an independent risk factor for pneumothorax [3]. However, according to the results of the systematic review of Huo et al. the prone position should be preferred over the supine position with regard to lower pneumothorax risk. They identified the lateral decubitus position with biopsy of the dependent lung as the patient position with the lowest risk of pneumothorax [17]. This position is not routinely used at our institution and was therefore not included in the analysis. Eventually these contrary results reflect the fact, that the exact mechanism how body position affects the pneumothorax rate is not clearly understood. Besides pneumothorax, the rare but very serious complication of systemic air embolism, which is influenced by patient position (prone patient position has been associated with a higher risk for air embolism [23]), should also be taken into account for procedure planning.

Limitations of our study are the retrospective study design with only a limited number of patients. It is not possible to overcome the potential bias due to interventionalist’s preference for opioid application and choice of dosage. Moreover, our study results do not allow for any dosing recommendations. There may be a selection bias due to the tertiary referral center setting. The study setting has potentially led to a disproportionate selection of high-risk patients, thus affecting the overall representativeness of the study sample. Some other potentially relevant risk factors, such as the puncture angle or operator experience, were not analyzed in this study. Lastly, the reason for the significant impact of body position on the incidence of pneumothorax remains unexplained.

## Conclusion

Our study results show that periinterventional analgosedation with the opioid piritramide may reduce the pneumothorax rate in CT-PCB of the lung. In contrast, prone patient position and repeated pleural passages increase the risk for an iatrogenic pneumothorax.

## Abbreviations

**CI**: Confidence interval
**CIRSE**: Cardiovascular and interventional radiological society of Europe
**CT**: Computed tomography
**CT-PCB**: CT-guided percutaneous core biopsy
**G**: Gauge
**OR**: Odds ratio
**SD**: Standard deviation
